# Supplementary figures and images for: Stack of cellular lamellae forms a silvered cortex to conceal the opaque organ in a transparent gastropod in epipelagic habitat
Source: PeerJ. 2022 Oct 28;10:e14284. doi: 10.7717/peerj.14284 (PMC9620974; doi:10.7717/peerj.14284)

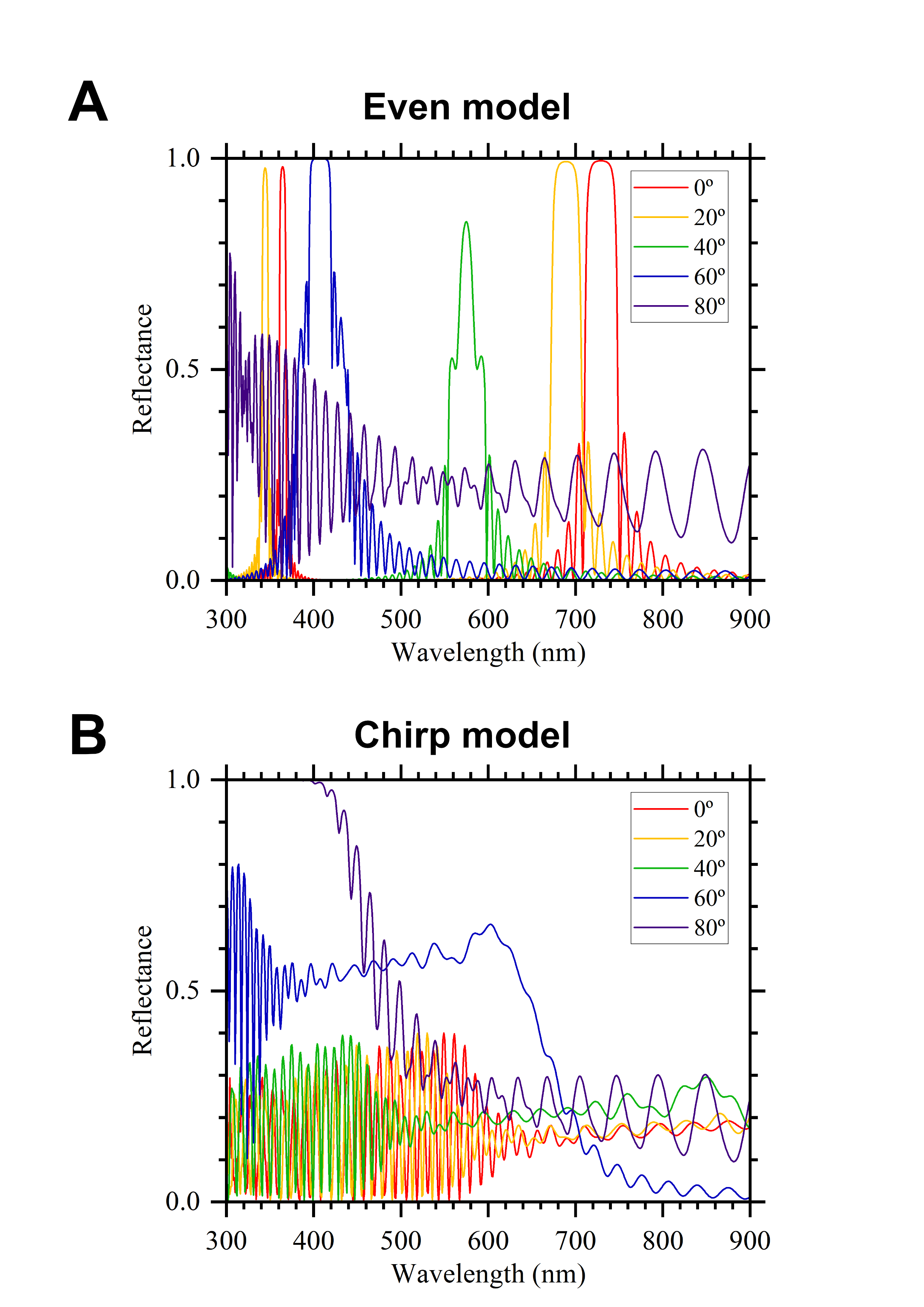

Supplement: Supplemental Information 3 — Reflectance values are the sum of TE and TM waves. [file peerj-10-14284-s003.png]

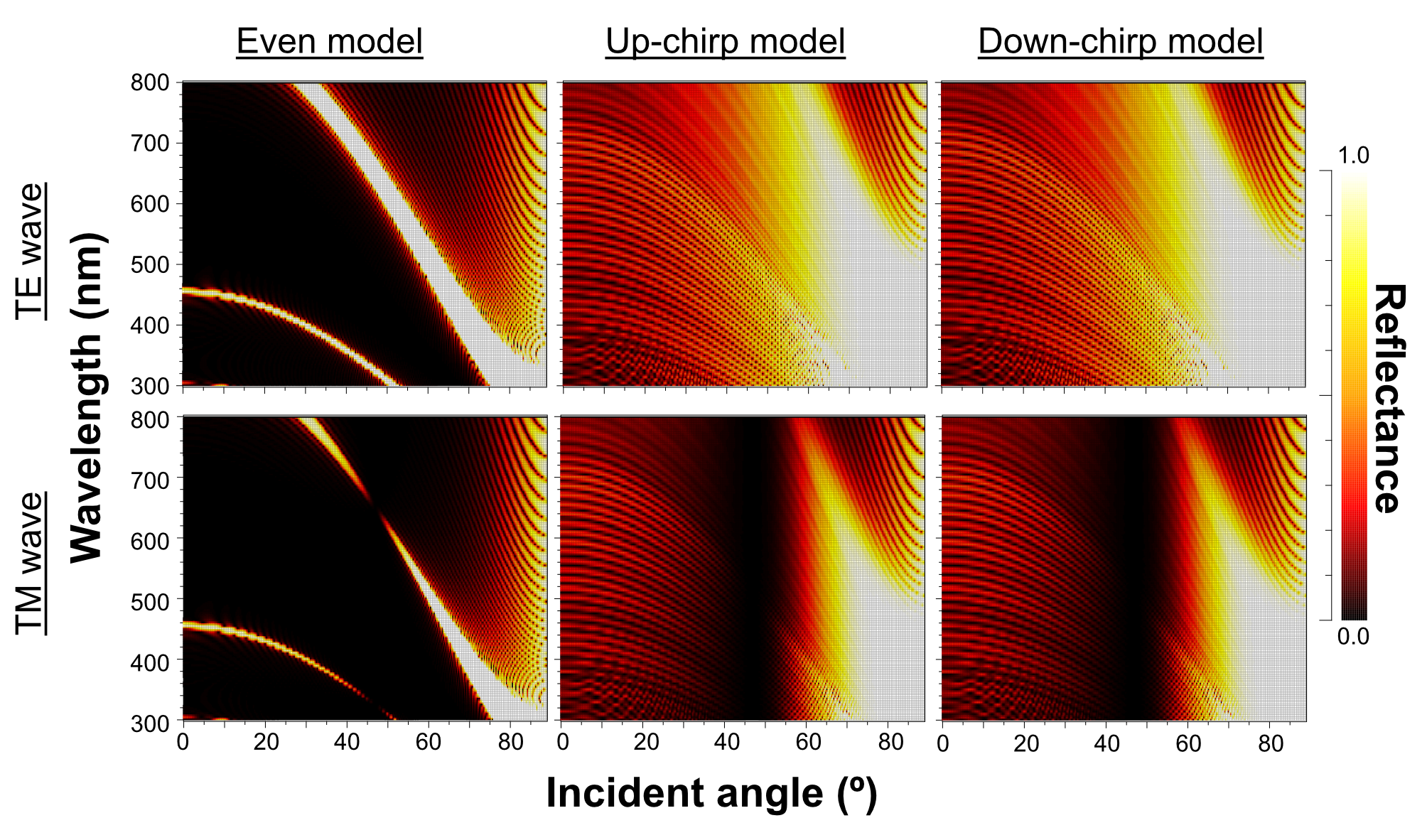

Supplement: Supplemental Information 4 — Even model (left), up-chirp model (middle), and down-chirp model (right) for TE wave (upper row) and TM wave (lower row). The difference in refractive index between the lamella and space ( Δn) is 0.1 in these simulations. No reflection occurs at approximately 47° (Brewster’s angle) for TM. [file peerj-10-14284-s004.png]
